# Does vascular endothelial growth factor (VEGF) predict local relapse and survival in radiotherapy-treated node-negative breast cancer?

**DOI:** 10.1038/sj.bjc.6690755

**Published:** 1999-10

**Authors:** B Linderholm, B Tavelin, K Grankvist, R Henriksson

**Affiliations:** Departments of Oncology, Clinical Chemistry, Umeå University Hospital, Umeå, Sweden

**Keywords:** VEGF, node-negative breast carcinoma, radiotherapy, relapse, survival

## Abstract

The aim of this study was to determine the association of vascular endothelial growth factor (VEGF) content in 302 consecutive node-negative breast cancer (NNBC) patients treated with only locoregional radiotherapy to relapse free- (RFS) and overall survival (OS). VEGF content in tumour cytosols was measured by an enzymatic immunoassay for the major isoform VEGF_165_. The median age was 56 years, the median follow-up time 56 months. A wide range (0.01–144.79 pg μg^−1^ DNA) of VEGF content was found (median 1.92). Significant associations were found between VEGF and oestrogen receptor (ER) content, progesterone receptor (PR) and tumour size (*P* = 0.005). Univariate analysis displayed significant reduced RFS and OS for patients with higher VEGF content (*P* = 0.0113 and *P* = 0.0075 respectively). A total of 43 recurrences have been found (ten local relapses within the breast, five in the axillary or supraclavicular lymph nodes and 28 distant metastasis). There was no significant correlation between the localization of the relapse and the VEGF content. Multivariate analysis suggested VEGF as the only predictor of OS (relative risk (RR) = 3.6, 95% confidence interval (CI) = 0.97–13.37), and in patients with T1 tumours (*n* = 236) the multivariate analysis clearly displayed VEGF as the only independent predictor of both RFS and OS (RR = 5.1, CI = 1.07–24.59). In the sub-group with ER-positive tumours (*n* = 229), multivariate analysis showed VEGF as the only significant predictor of RFS and OS (RR = 10.44, CI = 1.26–86.38). The results suggest VEGF_165_ as a predictor of RFS and OS in NNBC patients treated with locoregional radiotherapy, comprising especially patients with favourable prognosis of T1 tumours, or ER-positive tumours. The high VEGF expression might define a radioresistant phenotype, or indicate an early distant spread which might require adjuvant systemic treatment. © 1999 Cancer Research Campaign


*British Journal of Cancer* (1999) **81**(4), 727–732
© 1999 Cancer Research Campaign
Article no. bjoc.1999.0755

### Does vascular endothelial growth factor (VEGF) predict local relapse and survival in radiotherapy-treated node-negative breast cancer?

**B Linderholm[1], B Tavelin[1], K Grankvist[2] and R Henriksson[1]**

Departments of [1]Oncology and [2]Clinical Chemistry, Umeå University Hospital, Umeå, Sweden

**Summary** The aim of this study was to determine the association of vascular endothelial growth factor (VEGF) content in 302 consecutive node-negative breast cancer (NNBC) patients treated with only locoregional radiotherapy to relapse free- (RFS) and overall survival (OS). VEGF content in tumour cytosols was measured by an enzymatic immunoassay for the major isoform $VEGF_{165}$. The median age was 56 years, the median follow-up time 56 months. A wide range (0.01–144.79 pg $\mu g^{-1}$ DNA) of VEGF content was found (median 1.92). Significant associations were found between VEGF and oestrogen receptor (ER) content, progesterone receptor (PR) and tumour size ($P = 0.005$). Univariate analysis displayed significant reduced RFS and OS for patients with higher VEGF content ($P = 0.0113$ and $P = 0.0075$ respectively). A total of 43 recurrences have been found (ten local relapses within the breast, five in the axillary or supraclavicular lymph nodes and 28 distant metastasis). There was no significant correlation between the localization of the relapse and the VEGF content. Multivariate analysis suggested VEGF as the only predictor of OS (relative risk (RR) = 3.6, 95% confidence interval (CI) = 0.97–13.37), and in patients with T1 tumours ($n = 236$) the multivariate analysis clearly displayed VEGF as the only independent predictor of both RFS and OS (RR = 5.1, CI = 1.07–24.59). In the sub-group with ER-positive tumours ($n = 229$), multivariate analysis showed VEGF as the only significant predictor of RFS and OS (RR = 10.44, CI = 1.26–86.38). The results suggest $VEGF_{165}$ as a predictor of RFS and OS in NNBC patients treated with locoregional radiotherapy, comprising especially patients with favourable prognosis of T1 tumours, or ER-positive tumours. The high VEGF expression might define a radioresistant phenotype, or indicate an early distant spread which might require adjuvant systemic treatment. © 1999 Cancer Research Campaign

**Keywords:** VEGF; node-negative breast carcinoma; radiotherapy; relapse; survival

Post-operative adjuvant radiotherapy, delivered to patients with localized node-negative breast carcinoma (NNBC), has the aim to eradicate any micrometastases that may still be present in the breast after surgery (Veronesi et al, 1981; Fisher et al, 1989; Liljegren et al, 1994). However, there is a significant number of patients (25–30%) with only locally treated NNBC in which the tumour relapsed (McGuire, 1988). There are also a subgroup of patients that displayed an increased morbidity and even hampered survival after radiotherapy, dependent on suboptimal techniques used (Cuzick et al, 1994). Nevertheless, there is still a need to define more properly the subgroup of patients in whom more extensive therapy could be considered, in order to further optimize the radiotherapy given. Previously, a diversity of various factors related to tumours, such as hormone receptors, have shown to be of value when initiating systemic treatment to patients at risk for recurrence (Sigurdsson et al, 1990). Tumour vascularization, measured as microvessel density, has been proposed to be of independent prognostic value in breast carcinoma patients, with a worse outcome for patients with a higher vascularity in their primary tumours (Weidner et al, 1991; Bosari et al, 1992; Horak et al, 1992; Gasparini et al, 1993,1994; Toi et al, 1993; Fox et al, 1994; Obermair et al, 1995). On the other hand, radiotherapy requires adequate blood supply, and is suggested as less efficient in tissues with poor vascularization (Hobson and Denekamp, 1984). As one of the most potent growth factors known, VEGF induces endothelial cell proliferation and migration, increases vascular permeability, and co-function with proteolytic enzymes involved in tumour invasiveness (Ferrara et al, 1989; Lindgren et al, 1997). A high correlation is suggested between microvessel density and the expression of vascular endothelial growth factor (VEGF) (Toi et al, 1996). Recently, we and others proposed VEGF content to be a predictor of overall survival (OS) in primary breast carcinoma (Gasparini et al, 1997; Eppenberg et al, 1998; Linderholm et al, 1998).

This study aimed to determine the predictive value of VEGF for relapse-free survival (RFS) and overall survival (OS) in 302 consecutive node-negative patients treated with locoregional radiotherapy following conservative surgery. As far as we have found, no other controlled evaluation has been undertaken to specifically define the prognostic value of vascularization or VEGF with regard to radiotherapy and survival.

*Received 29 January 1999*
*Revised 31 March 1999*
*Accepted 20 April 1999*

*Correspondence to:* R Henriksson

### MATERIALS AND METHODS

### Patient data

Clinical information and tumour samples were collected from 302 consecutive unselected women with invasive node-negative breast carcinoma (NNBC), diagnosed and primarily treated for localized tumour between 1990 and 1995 in the health care region of

**Table 1** Clinicopathologic characteristics of the patients

| Variables | No. of patients | |
|---|---|---|
| Patients enrolled | 302 | |
| Histological type | | |
| Ductal invasive | 258 | |
| Lobular invasive | 14 | |
| Others | 30 | |
| Tumour size | | |
| T1 | 236 | |
| T2 | 66 | |
| Histopathological grading | | |
| I | 29 | |
| II | 113 | |
| III | 95 | |
| Not analysed | 65 | |
| Oestrogen receptor (ER) | | |
| pos (≥ 0.1 fmol $\mu g^{-1}$ DNA) | 229 | |
| neg (< 0.1 fmol $\mu g^{-1}$ DNA) | 73 | |
| VEGF levels (pg $\mu g^{-1}$ DNA) | | |
| All patients (median, range) | 302 | 1.92 (0.01–144.79) |

northern Sweden. The median age was 56 years (range 29–74), and the median follow-up time for survivors was 56 months (range 22–91 months). The patients included had histologically verified invasive unilateral breast carcinoma without axillary lymph node involvement, or detectable distant metastasis (T1–2, N0, M0). A total of 236 patients had T1 tumours. Tumour classification and staging was in accordance with the International Union Against Cancer tumour-node-metastasis (UICC-TNM) classification. Primary treatment was given according to the guidelines of the North Swedish Breast Cancer Group. All patients were treated with segmental resection with axillary dissection. In the group with T2 tumours (21–50 mm) ($n$ = 66) no upper limit concerning the maximum size of the tumour was set for treatment with sector resection. Surgery was followed by radiotherapy delivered daily at 2-Gy fractions, 5 days per week to a total dose of 56 Gy. Radiotherapy was started within 4–6 weeks after surgery. The axilla were not included in the target volume. No patients received any adjuvant systemic treatment. The end points determined were local relapses, distant metastasis and survival. Patients with concomitant local failure and distant metastasis as first event were classified in the metastasis group. The number of patients for whom data were available varied among different prognostic factors studied, but in all cases tumour size, oestrogen receptor (ER), progesterone receptor (PR) and tumour cytosol VEGF protein were measured (Table 1). The analyses of tumour tissue were performed prior to radiotherapy and blindly to the clinical data.

### Tumour tissue preparation, VEGF and receptor analysis

During primary surgery, and after pathological examination, representative tumour tissue was cut out and frozen in liquid nitrogen until analysis. Frozen tumour tissue was homogenized in a microdish membrator (Braun, Melsungen, Germany) and suspended in cold standard receptor buffer (10 mM Tris pH 7.4, 1.5 mM EDTA, 10 mM sodium molybdate, 1.0 mM monothioglycerol). Supernatants were collected after 10 min refrigerated centrifugation at 20 000 ***g*** and used for analyses of steroid receptor and VEGF protein contents. The pelleted fractions were analysed for DNA content by the method of Burton, in order to evaluate cell concentrations in samples. A high correlation is observed between DNA content and protein content ($r$ > 0.90). The reason to choose DNA content for evaluating tumour cellularity in patients' samples for routine receptor analyses is to avoid measurements of extracellular proteins. DNA quantification has therefore been considered more specific than total protein concentration (Norgren et al, 1986).

VEGF was analysed using a quantitative immunoassay kit for human $VEGF_{165}$ (Quantikine, human VEGF, R & D Systems, Minneapolis, MN, USA). $VEGF_{165}$ is the most commonly found isoform of VEGF in both normal and transformed cells (Scott et al, 1998).

ER and PR content was determined by an enzyme immunoassay (Abbott Laboratories, Diagnostic Division, Abbott Park, IL, USA). Receptor concentration was expressed as fmol receptor per μg DNA, and tumours with a value lower than 0.1 fmol ER or PR $\mu g^{-1}$ DNA were considered as receptor-negative, those with a value equal to or higher than 0.1 fmol ER or PR $\mu g^{-1}$ DNA as receptor-positive.

### Statistical methods

Association between $VEGF_{165}$ content and earlier established prognostic or predictive factors were tested by the Pearson $\chi^2$ test. Survival was estimated using the Kaplan–Meier method, and comparison between study groups was performed with the log-rank test. The cut-off value used was the median level of VEGF in this group of patients. To evaluate the simultaneous effect on different factors on survival, the Cox proportional hazard model was used. The survival time was measured from date of diagnosis to the date of the first relapse or death. In all tests the significance level was set to 0.05, and all were two-sided tests.

### RESULTS

### Distribution of $VEGF_{165}$

The median value was 1.92 pg $\mu g^{-1}$ DNA; however, a wide quantitative range of cytosolic $VEGF_{165}$ protein was found (range 0.01–144.79). Among the other prognostic variables, VEGF (≤ 1.92 vs > 1.92 pg $\mu g^{-1}$ DNA) was associated with ER content (ER-positive vs -negative, $P$ < 0.002), PR content (PR-positive vs -negative, $P$ = 0.006) and tumour size (≤ 2 cm vs 2.1–5.0 cm, $P$ = 0.005). A borderline value was seen between VEGF and higher histological grade (grades I + II vs III, $P$ = 0.053). No association was found between VEGF and histopathological type (ductal vs lobular and others, $P$ = 0.192), or between VEGF and age (≤ 56 years vs > 56 years, $P$ = 0.730).

### Association between the site of first relapse and VEGF content

A total of 43 relapses were found. There were ten in-breast failures, five metastasis in the axillary or supraclavicular lymph nodes and 28 distant metastasis as first events. One patient had both local failure and visceral metastasis as first event and classified in the distant metastasis group. There was no statistically significant correlation between the localization of the first relapse and the VEGF content ($P$ = 0.773). In the group with local recurrence within the breast, 70% ($n$ = 7 of 10) had a VEGF content above the median value, compared to 80% ($n$ = 4 of 5) in the group with

 *© 1999 Cancer Research Campaign*

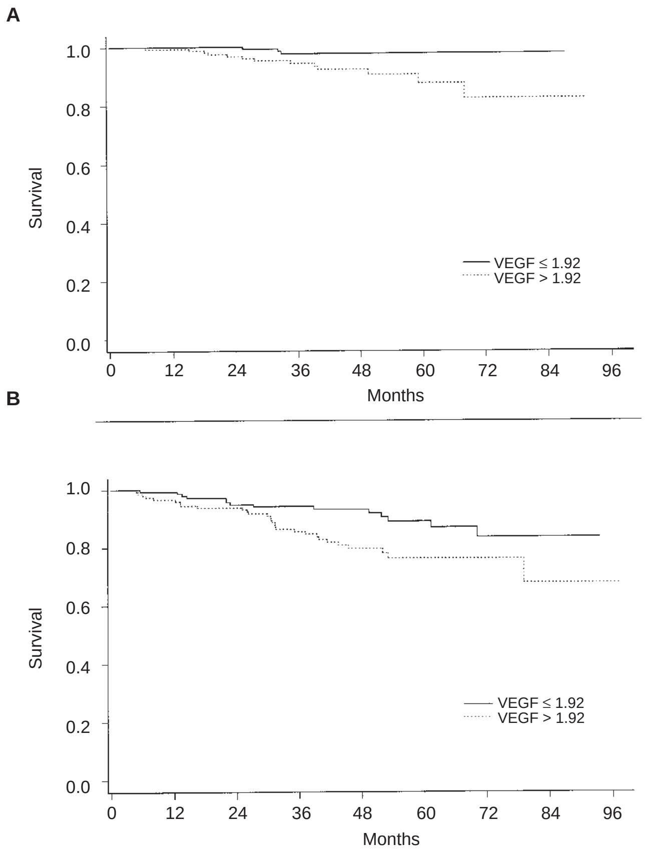

**Figure 1** The probability of (**A**) relapse-free survival ($P = 0.0113$) and (**B**) overall survival ($P = 0.0075$) for 302 node-negative patients primary treated with conservative surgery followed by radiotherapy according to vascular endothelial growth factor (VEGF). The cut-off value used is the median level in this group (1.92 pg μg$^{-1}$ DNA)

lymph node metastasis and 64% ($n$ = 18 of 28) in patients with distant metastasis.

### Univariate analysis

A statistically significant difference in RFS and OS (≤ 1.92 vs > 1.92 pg μg$^{-1}$ DNA), was found, with reduced survival times for patients with a higher level of cytosolic VEGF ($P = 0.0113$, Figure 1A; and $P = 0.0075$, Figure 1B respectively). In addition to VEGF, histological grade (I + II vs III) ($P = 0.0158$) and tumour size (T1 vs T2) ($P = 0.0020$) were statistically significant for RFS, while ER status (positive vs negative) ($P = 0.9579$), histological type (ductal vs lobular and others) ($P = 0.6205$) and age (≤ 56 years vs > 56 years) ($P = 0.1580$) were not. Moreover, histological grade and ER status were statistically significant for OS in univariate analyses ($P = 0.0496$ and $P = 0.0286$ respectively). Morphological features ($P = 0.7300$), tumour size ($P = 0.6869$) and age ($P = 0.7738$) were not significant for OS in this material. Univariate analysis in the subgroup with T1 tumours ($n = 236$) showed statistically significant reductions in RFS ($P = 0.0007$) and OS ($P = 0.0065$) for patients with VEGF content above the median value 1.92 pg μg$^{-1}$ DNA. Univariate analysis in the sub-group with ER-positive tumours ($n = 229$) showed VEGF as a predictive factor for RFS ($P = 0.0012$) and OS ($P = 0.0006$), with reduced survival times for patients with VEGF content above the median value (1.92 pg μg$^{-1}$ DNA).

### Multivariate analysis

Analysis of the joint effect of combining VEGF determination with the other prognostic factors, and age in order to avoid an effect of age-related mortality, proposed VEGF as the only valuable predictor of overall survival ($P = 0.0554$; confidence interval (CI) = 0.97–13.4). Patients with higher VEGF values in tumours (> 1.92 pg μg$^{-1}$ DNA) had a 3.62 times increased risk of death than those with lower VEGF values. The other included factors, tumour size, ER content, histological grade and age failed to retain as independent predictors for OS in multivariate analysis (Table 2).

For RFS, histological grade was the only independent predictive factor ($P = 0.0289$; relative risk (RR) = 2.29, CI = 1.09–4.81), while VEGF failed as an independent predictor of RFS ($P = 0.1406$), though still with an increased risk for recurrence of 1.77. Tumour size, ER status and age were not significant predictors of RFS.

The results from multivariate analysis in the group with only T1 tumours ($n = 236$), showed VEGF as the only independent predictor of RFS ($P = 0.0038$, RR = 5.18, CI = 1.70–17.78) and OS ($P = 0.0313$, RR = 5.62, CI = 1.17–27.27), while the other factors included failed (Table 3). The results from multivariate analysis in the sub-group with ER-positive tumours ($n = 229$), showed histological grade ($P = 0.0192$, RR = 2.71, CI = 1.18–6.24) and VEGF ($P < 0.0402$, RR = 248, CI = 1.04–5.93) as independent predictors

**Table 2** Multivariate Cox regression analysis on relapse free (RFS) and overall survival (OS) in all 302 node-negative breast cancer patients treated with segmental resection + radiotherapy

| | RFS | | | OS | | |
|---|---|---|---|---|---|---|
| **Variable** | **RR** | **95% CI** | ***P*** | **RR** | **95% CI** | ***P*** |
| VEGF | | | | | | |
| ≤ 1.92 vs >1.92 pg μg$^{-1}$ DNA | 1.77 | 0.83–3.79 | 0.1406 | 3.62 | 0.97–13.5 | 0.0554 |
| Estrogen receptor | | | | | | |
| ER– vs ER+ | 1.38 | 0.59–3.21 | 0.4533 | 2.06 | 0.68–6.17 | 0.1988 |
| Tumour size | | | | | | |
| T1 vs T2 | 1.99 | 0.95–4.19 | 0.0682 | 1.08 | 0.27–2.94 | 0.8692 |
| Histopathological grade | | | | | | |
| I+II vs III | 2.29 | 1.09–4.81 | 0.0289 | 1.95 | 0.62–6.13 | 0.2530 |
| Age, years | | | | | | |
| ≤ 56 vs > 56 | 1.03 | 0.50–2.12 | 0.9335 | 1.03 | 0.37–2.92 | 0.9437 |

**Table 3** Multivariate Cox regression analysis on relapse-free (RFS) and overall survival (OS) in 236 patients with T1 node-negative breast cancer tumours

| | RFS | | | OS | | |
|---|---|---|---|---|---|---|
| **Variable** | **RR** | **95% CI** | ***P*** | **RR** | **95% CI** | ***P*** |
| VEGF | | | | | | |
| ≤ 1.92 vs >1.92 pg μg⁻¹ DNA | 5.18 | 1.70–17.78 | 0.0038 | 5.62 | 1.17–27.27 | 0.0313 |
| Estrogen receptor | | | | | | |
| ER– vs ER+ | 1.44 | 0.46–4.53 | 0.5325 | 1.71 | 0.45–6.40 | 0.4254 |
| Histopathological grade | | | | | | |
| I+II vs III | 1.89 | 0.74–4.83 | 0.1854 | 1.57 | 0.41–5.91 | 0.5075 |
| Age, years | | | | | | |
| ≤ 56 vs > 56 | 1.97 | 0.79–4.89 | 0.1434 | 2.37 | 0.68–8.25 | 0.1727 |

**Table 4** Multivariate Cox regression analysis on relapse-free survival (RFS) overall survival (OS) in 229 patients with oestrogen receptor-positive node-negative breast cancer tumours

| | RFS | | | OS | | |
|---|---|---|---|---|---|---|
| **Variable** | **RR** | **95% CI** | ***P*** | **RR** | **95% CI** | ***P*** |
| VEGF | | | | | | |
| ≤ 1.92 vs >1.92 pg/μg DNA | 2.48 | 1.04–5.93 | 0.0402 | 10.44 | 1.26–86.4 | 0.0296 |
| Tumour size | | | | | | |
| T1 vs T2 | 2.02 | 0.85–4.80 | 0.1128 | 0.56 | 0.68–4.60 | 0.5902 |
| Histopathological grade | | | | | | |
| I+II vs III | 2.71 | 1.18–6.24 | 0.0192 | 2.67 | 0.63–11.26 | 0.1808 |
| Age, years | | | | | | |
| ≤ 56 vs > 56 | 1.14 | 0.50–2.62 | 0.7435 | 1.30 | 0.32–5.26 | 0.7159 |

of RFS. For OS, VEGF was the only significant predictor ($P$ = 0.0296, RR = 10.44, CI = 1.26–86.39), while the other factors included failed (Table 4).

### DISCUSSION

The hypothesis to be tested in this study was specifically to explore the possibility that VEGF, as an indicator of the degree of vascular activity, could predict the efficacy of locoregional radiotherapy in breast carcinoma after conservative surgery. Radiotherapy is known to reduce local recurrence, and recently has also been proposed to enhance survival (Overgaard et al, 1997). The present results from 302 consecutively sampled tumours indicate that $VEGF_{165}$ could be of value as a predictor of both RFS and OS in women subjected to locoregional therapy for breast carcinoma. The correlation was especially seen in patients in general considered to have a good prognosis, i.e. T1 tumours or ER-positive tumours, with an increased risk of recurrence and death of 5.18 and 2.48 (RFS) and 5.62 and 10.44 (OS) respectively. In these sub-groups we could thus identify patients with a high risk of recurrence or death, and consequently surgery followed by radiotherapy is not sufficient.

However, it has to be emphasized that with regard to RFS, VEGF was found as a non-significant factor, but with an increased risk of recurrence of 1.77. Still, multivariate analysis showed VEGF as an independent predictor of overall survival with a 3.6 times increased risk of death for patients with higher $VEGF_{165}$ content. Having in mind that this study had relatively short follow-up (56 months) and few events, 43 recurrences and 16 deaths, prolonged follow-up may change the results. It has to be stressed that other factors such as local or systemic therapy delivered after relapses could influence the survival times. It has been shown that tumours with a high angiogenic activity, measured by counting vessel density, seem to be less sensitive to endocrine treatment or chemotherapy than tumours with lower vessel density (Gasparini et al, 1995, 1996). Although women with local relapses have an increased risk of distant metastases, the 5-year survival rate has been reported as close to 70%. In a large study consisting of more than 2000 node-negative patients treated with quadrantectomy followed by radiotherapy, the only factor associated with an increased risk of local relapse, not of distant metastasis, was an extensive intraductal component (Veronesi et al, 1995). Moreover, in this study low histological grade was the strongest factor for RFS, but not of significance for OS, which is in accordance with our results.

Due to different splicing, VEGF exists in at least four different isoforms with 121, 189 and 206 amino acids ($VEGF_{121}$, $VEGF_{165}$, $VEGF_{189}$ and $VEGF_{206}$ respectively), which have different affinities to heparin. $VEGF_{165}$ is the predominant isoform secreted by a variety of both normal and transformed cells. A significant proportion remains bound to the cell surface and the extracellular matrix. For those reasons, we chose to measure $VEGF_{165}$. Moreover, $VEGF_{165}$ has been reported as the major protein isoform, despite the fact that mRNA from also $VEGF_{121}$ and $VEGF_{189}$ are present in human breast carcinoma (Scott et al, 1998).

The effects of irradiation are known to be, at least partially, dependent on oxygen tension and thus the vascular supply to tumours (Hobson and Denekamp, 1984; Folkman, 1990). Higher levels of VEGF have been reported in 'normal' tumour-adjacent tissue than in breast tissue in the contralateral breast and this might imply a higher vascularization and subsequently increased radiosensitivity (Schlaeppi et al, 1996). On the other hand,

*© 1999 Cancer Research Campaign*

hypoxia has shown to up-regulate VEGF, both at mRNA and protein level, in tumour cell lines and around the necrotic foci of tumours (Shweiki et al, 1992; Scott et al, 1998). It has also earlier been reported a high correlation between microvessel density and the expression of VEGF (Toi et al, 1996). Nevertheless, paradoxically or not, the present observation in contrary showed that a higher content of VEGF is associated with a reduced RFS and OS following radiotherapy. Patients with a higher VEGF expression seem to be more likely to have a local recurrence or to develop distant metastasis. No significant correlation was found between the increased VEGF content and the site of the first metastasis. However, the number of events were too small for definite conclusions. The results find support in previous studies that showed microvessel density and VEGF content to be a predictor of survival in breast carcinoma patients, regardless of primary and adjuvant systemic treatment (Bosari et al, 1992; Gasparini et al, 1993; Toi et al, 1993; Fox et al, 1994; Obermair et al, 1995; Linderholm et al, 1998).

We thus conclude that VEGF content in the primary tumour might be a predictor of relapse-free and, most important, overall survival in node-negative breast cancer treated by locoregional radiotherapy. The high VEGF content associated to a worse outcome might be a result of a radioresistant phenotype, or an early disseminated disease which requires adjuvant systemic therapy. These issues deserve further studies.

### ACKNOWLEDGEMENTS

This study was supported by grants from Lion’s Cancer Research Foundation, Umeå, and the Swedish Cancer Foundation

### REFERENCES

Bosari S, Lee AKC, De Lellis RA, Wiley BD, Heatley QJ and Silverman ML (1992) Microvessel quantitation and prognosis in invasive breast carcinoma. *Hum Pathol* **23**: 755–761

Cuzick J, Stewart H, Rutqvist L, Houghton J, Edwards R, Redmond C, Peto R, Baum M, Fisher B and Host H (1994) Cause-specific mortality in long-term survivals of breast cancer who participate in trials of radiotherapy. *J Clin Oncol* **12**: 447–453

Eppenberger U, Kueng W, Schlaeppi J-M, Roesel JL, Benz C, Mueller H, Matter A, Zuber M, Luescher K, Litschgi M, Schmitt M, Foekens JA and Eppenberger-Castori S (1998) Markers of tumor angiogenesis and proteolysis independently define high- and low-risk subsets of node-negative breast cancer patients. *J Clin Oncol* **16**: 3129–3136

Ferrara N and Henzel WJ (1989) Pituitary follicular cells secrete a novel heparin-binding growth factor specific for vascular endothelial cells. *Biochem Biophys Res Commun* **161**: 851–858

Ferrara N, Houck K, Jakeman L and Leung DW (1992) Molecular and biological properties of the vascular endothelial growth factor family of proteins. *Endocr Rev* **1**: 18–32

Fisher B, Redmond C, Poisson R, Margolese R, Wolmark N, Wickerman L, Fisher E, Deutsch M, Caplan R and Pilch Y (1989) Eight-years results of a randomized clinical trial comparing total mastectomy and lumpectomy with or without irradiation in the treatment of the breast. *N Engl J Med* **320**: 822–828

Folkman J (1990) What is the evidence that tumors are angiogenesis dependent? *J Natl Cancer Inst* **82**: 4–6

Fox SB, Leek RD, Smith K, Hollyer J, Greenall M and Harris AL (1994) Tumour angiogenesis in node-negative breast carcinomas – relationship to epidermal growth factor receptor, estrogen receptor, and survival. *Breast Cancer Res Treat* **29**: 109–116

Gasparini G, Pozza F and Harris AL (1993) Evaluating the potential usefulness of new prognostic and predictive indicators in node-negative breast cancer patients. *J Natl Cancer Inst* **85**: 1206–1219 (review)

Gasparini G, Weidner N, Bevilaqua P, Maluta S, Dalla Palma P, Caffo O, Barbareschi M, Boracchi P, Marubini E and Pozza F (1994) Tumour microvessel density, p53 expression, tumour size and peritumoural lymphatic vessel invasion are relevant prognostic markers in node-negative breast carcinoma. *J Clin Oncol* **12**: 454–466

Gasparini G, Barbareschi M, Boracchi P, Verderio P, Caffo O, Meli S, Dalla Palma P, Marubini E and Bevilacqua P (1995) Tumor angiogenesis predicts clinical outcome of node-positive breast cancer patients treated with adjuvant hormone therapy or chemotherapy. *Int J Cancer* **1**: 131–141

Gasparini G, Fox SB, Verderio P, Bonoldi E, Bevilacqua P, Boracchi P, Dante S, Marubini E and Harris AL (1996) Determination of angiogenesis adds information to estrogen receptor status in predicting efficacy of adjuvant tamoxifen in node-positive breast cancer patients. *Clin Cancer Res* **2**: 1191–1198

Gasparini G, Toi M, Gion M, Verderio P, Dittadi R, Hanatani M, Matsubara I, Vinante O, Banddi E, Boracchi P, Gatti C, Suzuki H and Tominaga T (1997) Prognostic significance of vascular endothelial growth factor protein in node-negative breast carcinoma. *J Natl Cancer Inst* **89**: 139–147

Hobson B and Denekamp J (1984) Endothelial proliferation in tumours and normal tissues: continuous labelling studies. *Br J Cancer* **49**: 405–413

Horak ER, Leek R, Klenk, Lejeune S, Smith K, Stuart M, Greenall M and Harris AL (1992) Angiogenesis, assessed by platelet/endothelial cell adhesion molecule antibodies, as indicator of node metastasis and survival in breast cancer. *Lancet* **340**: 1120–1124

Houck KA, Ferrara N, Winer J, Cachianes G, Li B and Leung DW (1991) The vascular endothelial growth factor family: identification of a fourth molecular species and characterization of alternative splicing of RNA. *Mol Endocrinol* **12**: 1806–1814

Liljegren G, Holmberg L, Adami H-O, Westman G, Graffman S and Bergh J (1994) The Uppsala-Örebro Breast-Cancer Study Group. Sector resection with or without postoperative radiotherapy for stage I breast cancer; five year results of a randomised trial. *J Natl Cancer Inst* **86**: 717–722

Linderholm B, Tavelin B, Grankvist, K and Henriksson R (1998) Vascular endothelial growth factor ($VEGF_{165}$) is of high prognostic value in node-negative breast carcinoma. *J Clin Onc* 16;**9**: 3121–3128

Lindgren M, Johansson M, Sandström J, Jonsson Y, Bergenheim AT and Henriksson R (1997) VEGF and tPA co-expressed in malignant glioma. *Acta Oncol* **36**; 6: 615–618

McGuire WL (1988) Adjuvant therapy of node-negative breast cancer. Another point of view. *J Natl Cancer Inst* **80**: 1075–1076

Norgren A, Fernö M and Borg Å (1986) Observations on wet weight, protein and DNA as reference for steroid receptors in malignant mammary tumours. *Anticancer Res* **6**: 59–64

Obermair A, Kurz C, Czerwenka K, Thoma M, Kaider A, Wagner T, Qitsch Q and Sevelda P (1995) Microvessel density and vessel invasion in lymph-node-negative breast cancer: effect on recurence-free survival. *Int J Cancer* **62**: 126–131

Overgaard M, Hansen PS, Overgaard J, Rose C, Andersson M, Bach F, Kjaer M, Qadeberg CC, Mouridsen HT, Jensen MB and Zedeler K (1997) For the Danish Breast Cancer Cooperative Group 826 trial. Postoperative radiotherapy in high-risk or menopausal women with breast cancer who receive adjuvant chemotherapy. *N Engl J Med* **337**: 949–955

Schlaeppi JM, Eppenberger U, Martiny-Baron G and Küng W (1996) Chemiluminescence immunoassay for vascular endothelial growth factor (vascular permeability factor) in tumour tissue homogenates. *Clin Chem* **42**: 1777–1784

Scott PAE, Gleadle JM, Bicknell R and Harris AL (1998*a*) Role of the hypoxia sensing system, acidity and reproductive hormones in the variability of vascular endothelial growth factor induction in human breast carcinoma cell lines. *Int J Cancer* **75**: 706–712

Scott PAE, Smith K, Poulsom R, De Benedetti A, Bicknell R and Harris AL (1998*b*) Differential expression of vascular endothelial growth factor mRNA vs protein isoform expression in human breast cancer and relationship to eIF-4E. *Br J Cancer* **77**: 2120–2128

Shweiki D, Itin A, Soffer D and Keshet E (1992) Vascular endothelial growth factor induced by hypoxia may mediate hypoxia-initiated angiogenesis. *Nature* (Lond) **359**: 843–845

Sigurdsson H, Baldetorp B, Borg Å, Dahlberg M, Fernö M, Killander D and Olsson H (1990) Indicators of prognosis in node-negative breast cancer. *N Engl J Med* **332**: 1045–1053

Toi M, Hashitani J and Tominaga T (1993) Tumor angiogenesis is an independent prognostic indicator of primary breast carcinoma. *Int J Cancer* **55**: 341–374

Toi M, Kondo S, Suzuki H, Yamamoto Y, Inada K, Imazawa T, Taniguchi T and Tominaga T (1996) Quantitative analysis of vascular endothelial growth factor in primary breast cancer. *Cancer* **77**: 1101–1106

© 1999 Cancer Research Campaign 

Veronesi U, Saccozzi R, Del Vecchio M, Barfi A, Clemente C, DeLena M, Gallus Q, Greco M, Luini A, Marubini E, Musolino Q, Rilke F, Salvadori B, Zechini A and Zucali R (1981) Comparing radical mastectomy with quadrantectomy, axillary dissection and radiotherapy in patients with small cancers of the breast. *N Engl J Med* **305**: 6–11

Veronesi U, Marubini E, Del Vecchio M, Manzari A, Andreola S, Greco M, Luini A, Merson M, Saccozzi R, Rilke F and Salvadori B (1995) Local recurrences and distant metastsis after conservative breast cancer treatments: partly independent events. *Natl J Cancer Inst* **87**: 19–27

Weidner N, Semple JP, Welch WR and Folkman J (1991) Tumor angiogenesis and metastasis-correlation in invasive breast carcinoma. *N Engl J Med* **324**: 1–8

*© 1999 Cancer Research Campaign*
